# Force-Regulated Calcium Signaling of Lymphoid Cell RPMI 8226 Mediated by Integrin α_4_β_7_/MAdCAM-1 in Flow

**DOI:** 10.3390/biom13040587

**Published:** 2023-03-24

**Authors:** Dongshan Sun, Zhiqing Luo, Ying Kong, Ruiting Huang, Quhuan Li

**Affiliations:** 1 Institute of Biomechanics, School of Bioscience and Bioengineering, South China University of Technology, Guangzhou 510006, China; 2 Guangdong Provincial Engineering and Technology Research Center of Biopharmaceuticals, South China University of Technology, Guangzhou 510006, China

**Keywords:** integrin α_4_β_7_, MAdCAM-1, calcium signaling, RPMI 8226 cell line, flow chamber

## Abstract

MAdCAM-1 binds to integrin α_4_β_7_, which mediates the rolling and arrest of circulating lymphocytes upon the vascular endothelia during lymphocytic homing. The calcium response by adhered lymphocytes is a critical event for lymphocyte activation and subsequent arrest and migration under flow. However, whether the interaction of integrin α_4_β_7_ /MAdCAM-1 can effectively trigger the calcium response of lymphocytes remains unclear, as well as whether the fluid force affects the calcium response. In this study, we explore the mechanical regulation of integrin α_4_β_7_-induced calcium signaling under flow. Flou-4 AM was used to examine the calcium response under real-time fluorescence microscopy when cells were firmly adhered to a parallel plate flow chamber. The interaction between integrin α_4_β_7_ and MAdCAM-1 was found to effectively trigger calcium signaling in firmly adhered RPMI 8226 cells. Meanwhile, increasing fluid shear stress accelerated the cytosolic calcium response and enhanced signaling intensity. Additionally, the calcium signaling of RPMI 8226 activated by integrin α_4_β_7_ originated from extracellular calcium influx instead of cytoplasmic calcium release, and the signaling transduction of integrin α_4_β_7_ was involved in Kindlin-3. These findings shed new light on the mechano-chemical mechanism of calcium signaling in RPMI 8226 cells induced by integrin α_4_β_7._

## 1. Introduction

Lymphocyte homing plays an important role in adaptive immunity [1]. The high specificity of homing is accomplished by lymphocyte adherence to the vascular endothelium at target locations [1,2]. Lymphocyte homing is a multistep process comprising tethering, slow rolling, activation, arrest, and transmigration events that are controlled by homing molecules [3]. In this process, lymphocyte activation is of great importance to the functionality and development of lymphocytes [4].

Integrin α_4_β_7_, a homing receptor molecule, can mediate circulating lymphocytes to roll and arrest on the vascular endothelia of mucosal tissues by interacting with mucosal vascular address in cell adhesion molecule-1 (MAdCAM-1) [4,5,6,7]. During this process, intracellular calcium signals are generated [8]. Calcium signaling regulates several lymphocyte processes, including lymphocyte development, T cell and B cell activation, allergy, gene transcription, and effector functions [9,10]. The activation of immunoreceptors, including T cell, B cell, and Fc receptors, as well as chemokine and co-stimulatory receptors, results in an increase in intracellular Ca^2+^ levels [10]. Although integrin α_4_β_7_ is crucial for the adhesion and migration of circulating lymphocytes, the calcium response of lymphocytes resulting from the engagement of integrin α_4_β_7_ remains unclear [4].

Lymphocyte adhesion mediated by the interaction between integrin α_4_β_7_ and MAdCAM-1 is regulated by fluid force in the blood environment. Chemical interactions, as well as mechanical elements, can contribute to the regulation of the immune functions of leukocytes [11,12]. The activation of integrin α_4_β_7_ via the G-protein coupled receptor pathway is dependent on fluid shear stress [13], and the subsequent calcium signaling event is affected by flow force, such as the calcium response of T lymphocytes via the TCR/MHC-peptide [14] and the calcium bursting of leukocytes mediated by LFA-1/ICAM-1 [15,16]. Therefore, it is unclear whether the external force can regulate the calcium response triggered by integrin α_4_β_7_/MAdCAM-1.

Intracellular calcium release and extracellular calcium influx are the two primary mechanisms that increase intracellular Ca^2+^ levels in lymphocytes [9]. Calcium signaling triggered by integrin LFA-1 binding with ICAM-1 occurs due to the influx of calcium in neutrophils and HL-60 cells [15]. However, the identification of the pathway by which the cytoplasmic Ca^2+^ concentration is upregulated, and that which is involved in the calcium response mediated by integrin α_4_β_7_ remains unknown, as well as the key molecule that participates in this signaling pathway. To date, a number of adaptor proteins that serve as scaffolds for protein–protein interactions have been implicated in integrin outside-in signaling [17]. Kindlin family members are FERM domain-containing proteins that interact with the integrin β tails. Kindlin-3, a member of the Kindlin family specifically expressed in leukocytes, contributes to inside-out and outside-in signaling [18,19]. Kindlin-3 has been demonstrated to interact with LFA-1 to support the calcium response under flow; however, the activation of integrin α_4_β_7_ simulated by chemokine CCL25 combines with the dissociation of Kindlin-3 from the β tail of integrin α_4_β_7_ [13,15]. Thus, further study is required to clarify the role of Kindlin-3 in calcium signaling mediated by integrin α_4_β_7_.

In this study, we investigated the intercellular calcium response of RPMI 8226 cells, a lymphocyte model [20], mediated by integrin α_4_β_7_ using a system combining fluorescence microscopy and a parallel-plate flow chamber. The calcium signaling events triggered by the binding of integrin α_4_β_7_ to MAdCAM-1 under different flow shear stresses were described by the delay time, peak time, and peak calcium intensity. The results showed that calcium signaling in RPMI 8226 cells firmly adhered to integrin α_4_β_7_-MAdCAM-1 and was effectively activated and positively regulated by the concentration of immobilized MAdCAM-1 and fluid shear stress. Additionally, the extracellular calcium influx was associated with integrin α_4_β_7_-induced calcium signaling instead of intracellular calcium release. Moreover, Kindlin-3 was shown to be involved in the calcium signaling pathway produced by integrin α_4_β_7_. In conclusion, these findings provide new perspectives on cellular physiological processes at the molecular level.

## 2. Materials and Methods

### 2.1. Proteins and Cells

The recombinant human MAdCAM-1-Fc chimera (R&D Systems, Minneapolis, MN, USA), referred to as MAdCAM-1, is a disulfide-linked homodimer that contains the extracellular domain of human MAdCAM-1 and the Fc moiety of human IgG. The human plasmacytoma cell line RPMI 8226, which constitutively expresses integrin α_4_β_7_ as a receptor for MAdCAM-1, was obtained from the China Center for Type Culture Collection (CCTCC) (Wuhan, China). These cell lines were cultured in RPMI 1640 medium (Procell, Wuhan, China) supplemented with 10% fetal bovine serum (FBS) (Bioexplorer, Boulder, CO, USA) and antibiotics (Procell). Cells were grown in a 5% CO_2_ cell culture incubator (Thermo Fisher Scientific, Shanghai, China) at 37 °C.

### 2.2. Cell Transfection

The stable knockdown of Kindlin-3 was achieved using short hairpin RNA (shRNA). Kindlin-3 shRNA (5′-CCGAAUUGUACACGAGUAU-3′) was constructed using the pLKO.1-EGFP-puro vector containing anti-puromycin and EGFP labels (Tsingke, Beijing, China) [21]. Cells were seeded on the cell culture plate 1 d in advance and transfected with the plasmid at a cell confluence degree of 60–80% using the transfection reagent (Zeta-life, CA, USA) in accordance with the manufacturer’s guidelines. The cells were then screened with puromycin (Beyotime, Shanghai, China) (1 μg/mL) for approximately 3 weeks. After the screening, the cells were verified by flow cytometry combined with Western blotting to evaluate the effects of knockdown.

### 2.3. Measurement with Flow Cytometry and Western Blotting

To determine the fraction of Kindlin-3-silenced RPMI 8226 cells cultured in stably transfected cell lines, 5 × 10^5^ cells were harvested and washed twice in prechilled PBS (Gibco, Shanghai, China). The cells were resuspended in 2% BSA (*w*/*v*) (Sigma, Burlington, MA, USA) and incubated for 30 min. After centrifugation at 1000 rpm for 3 min, the cells were resuspended in PBS. Based on the EGFP coding sequence in the silencing plasmid, the cells were examined by flow cytometry (Bio-Rad, Hercules, CA, USA) to determine the proportion of Kindlin-3-silenced cells. Non-transfected cells were used as the negative controls.

The harvested cells were lysed in RIPA buffer (Sigma, Burlington, MA, USA), and the protein concentration was determined according to the bicinchoninic acid method using the BCA protein assay kit (Thermo Fisher Scientific, Rockford, IL, USA). Briefly, 100 μg of protein was separated by 8% SDS-PAGE gels per lane and transferred to nitrocellulose (NC) membranes (Yeasen, Shanghai, China). The membrane was blocked for 1 h with 5% skim milk before being incubated overnight at 4 °C with the primary antibodies against anti-Kindlin-3 (1:500; Solarbio, Beijing, China) or anti-β-actin (1:1000; CST, Danvers, MA, USA). The membrane was then incubated with secondary antibodies (1:3000; CST) conjugated with HRP (horse radish peroxidase) for 1 h at 25 °C, and the signal strength of the protein bands was detected using an enhanced chemiluminescence reagent (Thermo Fisher Scientific) and the gel documentation system (Clinx, Shanghai, China).

### 2.4. Functionalization of Flow Chamber Substrates

The flow chamber (working space: length × width × height = 2 × 0.5 × 0.0254 cm^3^) was functionalized using the method outlined in previous studies [11,22,23]. Initially, a coating zone (25 mm^2^) covered with clean silicon rubber was designated at the center of each cover slide (Thermo Fisher Scientific, Shanghai, China). Thereafter, MAdCAM-1 was diluted in 40 μL of solution, immediately added to the coating zone of the slide, and then incubated for 12 h in a refrigerator at 4 °C. After removing the MAdCAM-1, the slides were treated with HBSS containing 2% BSA (*w*/*v*) at 25 °C for 1 h to block non-specific adhesion. After more than 1 h of treatment with 2% BSA, the slides were equipped to the flow chamber for subsequent experiments, and the functionalization of the flow chamber was finalized. A variety of MAdCAM-1 concentrations were used and selected under different experimental conditions.

### 2.5. Loading with Calcium-Sensitive Dye and Treatment with Inhibitors

The procedure for loading calcium-sensitive dye into the cells was performed as previously reported, with some modifications [11,12,23]. The sensitive dye Fluo-4 AM ester (Invitrogen Life Technologies, Grand Island, NY, USA) was used to quantify the relative cellular calcium levels. Cells under good growth conditions were harvested and washed twice with HBSS. Loading buffer (2% BSA (*w*/*v*), 20 mmol/L glucose (Sigma, Burlington, MA, USA), and 20 mmol/L HEPES (Procell, Wuhan, China) in PBS) was used to resuspend the cells, which were then diluted to a density of 1 × 10^6^/mL. After incubating and gently vibrating the cells (10^6^/mL) for 30 min at 37 °C, Fluo-4 AM (2 μmol/L) was loaded into RPMI 8226 cells. After 3 min of centrifugation at 1000 rpm, the cells were then completely de-esterified from their intracellular AM ester by resuspending the cells in a loading buffer without dye at the same volume for 30 min more at 37 °C.

Additionally, to block the calcium release from the endoplasmic reticulum or calcium influx via the membrane calcium channel, the IP3 inhibitor 2-APB (100 μmol/L) was applied to RPMI 8226 cells for 8 min. Similarly, the membrane calcium channel inhibitor LaCl_3_ (μmol/L) was applied for 30 min. Alternatively, 0.1% DMSO (*w*/*v*) was applied as vehicle control.

### 2.6. Flow Chamber and Calcium Signaling Assays

RPMI 8226 cells at a density of 10^6^ µg/mL labeled with Fluo-4 AM were resuspended in imaging buffer containing 2% BSA (*w*/*v*), 10 mmol/L glucose, 10 mmol/L KCl (Damao, Tianjin, China), 1.5 mmol/L CaCl_2_ (Damao, Tianjin, China), 10 mmol/L MgCl_2_ (Damao, Tianjin, China), 30 mmol/L HEPES, and 110 mmol/L NaCl (Damao, Tianjin, China) at pH 7.35. Using a syringe pump (Harvard PHD22/2000; Harvard Apparatus, Holliston, MA, USA), the RPMI 8226 cells were perfused into a parallel-plate flow chamber to adhere to MAdCAM-1 substrate at different wall shear stresses.

**Figure 1:**
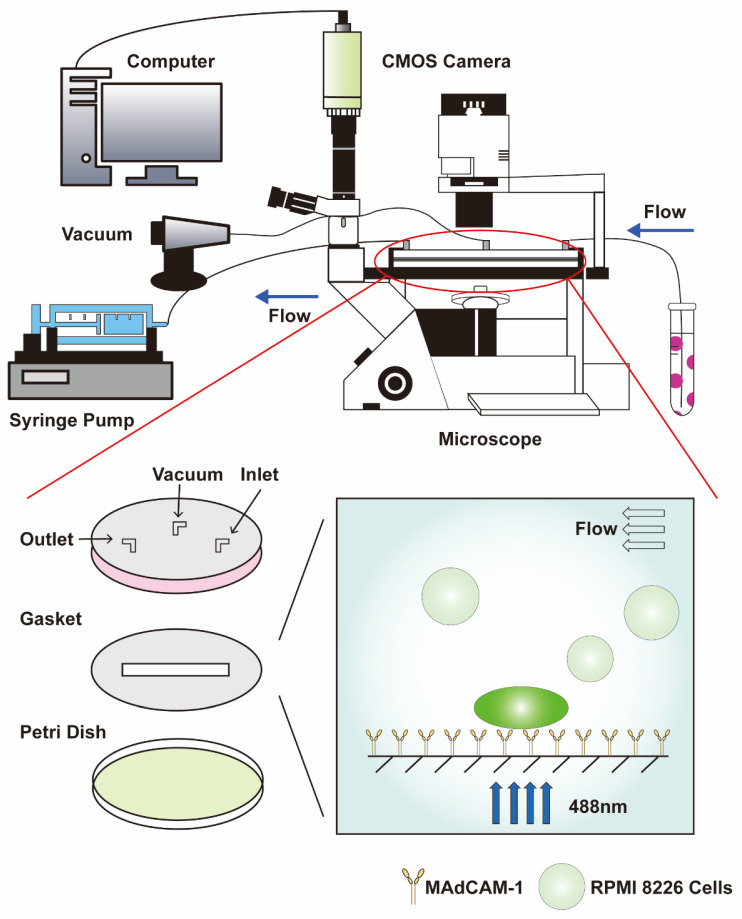
Schematic diagram of parallel plate flow chamber combined with a fluorescence detection system. The dark green cell represents RPMI 8226 cells that adhere stably to the substrate of MAdCAM-1, whose calcium signaling is activated. The light green cell only slides across the surface of the substrate and comprises the background of calcium signaling. CMOS: complementary metal oxide semiconductor.

For the specific adhesion assay, a CMOS camera (Nikon) (Nikon Corporation, Tokyo, Japan) was used to record the adhesion behavior at a frame frequency of 50 fps and an objective magnification of 10× (Figure 1). A firmly adhering cell was defined as a cell in which the travel displacement during 1 min was less than 10 μm. The number of firmly adhering cells within 7 min was estimated using Image-Pro Plus (version 6.0).

For the measurement of the calcium signaling of the cells, the fluorescence value of the firmly adhered cells was recorded using a CMOS camera and the software NIS-Elements AR 5.01.00 (Nikon) at a frame frequency of 20 fps, an exposure time of 50 ms, and an objective magnification of 40× (Figure 1). At least 15 typical calcium signaling events of the firmly adhering cells were collected among three repeat experiments for each group. Sequences were analyzed with the software NIS-Elements Analysis (version 6.0), and Microsoft Excel 2019 was used to extract time parameters and fluorescence intensity: F_IN_ = (F_IC_ − F_IB_)/F_IB_, which was used to normalize the fluorescence intensity of a firmly adherent cell, where F_IN_, F_IC_, and F_IB_ are the normalized cell fluorescence intensity, mean cell fluorescence intensity, and background fluorescence intensity, respectively, which is the mean of four fluorescence intensities from four equidistant round domains (36π µm^2^) around the cell at a distance of 24 µm [12].

The following three characteristics were used to describe calcium signaling in RPMI 8226 cells: the delay time, peak time, and peak calcium intensity of cell calcium bursting. The calcium bursting delay time was defined as the time interval between the onset of firm cellular adhesion at t = 0 and the subsequent sharp increase in cell fluorescence intensity. The peak time was defined as the duration of the rapid increase in cell fluorescence to the peak value after the plateau. The ratio of the normalized peak fluorescence intensity to the normalized plateau fluorescence intensity was used to calculate peak calcium intensity.

### 2.7. Statistical Methods

Statistical significance was determined using GraphPad Prism (version 7). Student’s *t*-test was used to confirm the statistical significance of the data in the two groups. Alternatively, one-way analysis of variance (ANOVA) was performed among various groups combined with Tukey’s test for multiple comparisons when the number of experimental groups was more than two.

## 3. Results

### 3.1. Calcium Bursting of RPMI 8226 Cells Adhered to MAdCAM-1 under Flow

**Figure 2:**
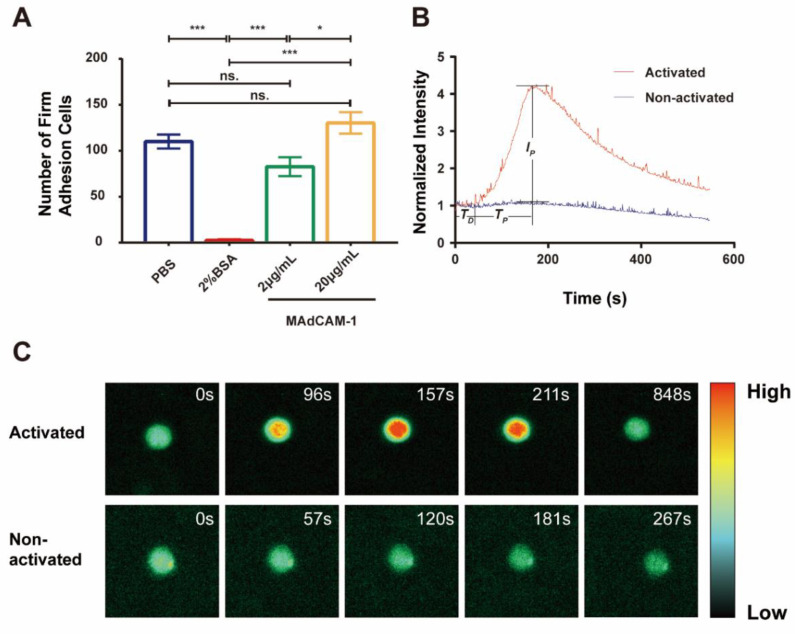
Calcium bursting of firmly adhered RPMI 8226 cells on MAdCAM-1 substrate under flow. (**A**) Number of firmly adhered RPMI 8226 cells on substrates coated with PBS, 2% BSA only, or plus MAdCAM-1 (2 and 20 μg/mL) under shear stress of 0.3 dyn/cm^2^. The data represent the mean ± standard error of the mean (SEM) from three independent experiments. The significance of the difference is shown by *p*-value, with ns. for *p* > 0.05, * for *p* < 0.05, and *** for *p* < 0.001. (**B**) The time course of the normalized fluorescence intensity of activated (red) or non-activated (blue) calcium signaling cells on the 20 μg/mL MAdCAM-1 substrate over the observation time. *T_D_*, delay time; *T_P_*, peak time; *I_P_*, normalized peak intensity. (**C**) Two series of fluorescence pseudo-color images are shown to illustrate the activated calcium signaling of firmly adhered RPMI 8226 cells and the non-activated one on 20 μg/mL MAdCAM-1 at different time points.

To investigate calcium signaling mediated by integrin α_4_β_7_ in RPMI 8226 cells under flow conditions, we evaluate the firm adhesion and calcium response of RPMI 8226 cells. Using a parallel plate flow chamber combined with a fluorescence detection system (Figure 1), the cells were found to adhere to the substrate coated with MAdCAM-1 at a wall shear stress of 0.3 dyn/cm^2^. Compared with those occurring on the blank substrates, firm adhesion events occurring on the substrates treated with 2% BSA were reduced significantly, suggesting that treatment with 2% BSA could block non-specific adhesion (Figure 2A, Movie S1). Moreover, the number of firmly adhering cells on the substrates coated with MAdCAM-1 plus 2% BSA increased and was positively correlated with the concentration (Figure 2A). To further demonstrate the specificity of α_4_β_7_ mediating cell adhesion, integrin α_4_ antibody (MAB1354) and integrin β_7_ antibody (MAB13544669) were incubated with the cells, and MAdCAM-1 antibody (F-6) was added to the substrate. As a result, a marked reduction in the number of firm adhesion cells was observed (Figure S1). These results indicate that the adhesion of RPMI 8226 cells to MAdCAM-1-coating substrates was specifically mediated by the interaction between MAdCAM-1 and its receptor integrin α_4_β_7_ expressed on RPMI 8226 cells.

To quantify the dynamic calcium signaling process, typical real-time normalized intensity curves were generated for the two RPMI 8226 cells (Figure 2B, Movie S2). When the cells were firmly adhered on 20 μg/mL MAdCAM-1 substrate, no significant change in the fluorescence intensity of the calcium response was observed during firm adhesion for non-activated cells, whereas a significant peak of the fluorescence intensity was observed in activated cells. The fluorescence intensity was initially maintained at a lower level for a duration of time, which then increased swiftly to its peak before gradually falling to its original level (Figure 2B,C, Movie S2). The calcium response of a firmly arresting cell occurred after a latent phase or delay time (*T_D_*), which represents the time gap between becoming firmly arrested and the calcium response. Meanwhile, the fluorescence intensity reached its maximum after increasing for a period of time (*T_P_*). The ratio of the highest fluorescence intensity to the average fluorescence intensity during the delay period was used to define the peak calcium intensity (*I_P_*), which represents the complete release of cytosolic calcium ions. The amount of cytosolic calcium released increased with peak calcium intensity. Five representative fluorescence images of activated and non-activated cells are shown in Figure 2C.

### 3.2. Concentration Dependence of Calcium Signaling of RPMI 8226 Cells

**Figure 3:**
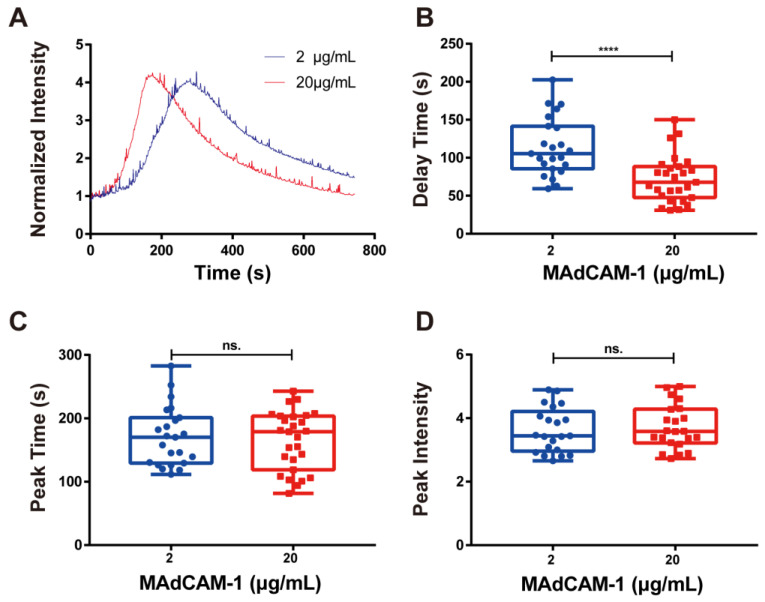
Concentration-dependent calcium signaling. (**A**) Time course, (**B**) delay time, (**C**) peak time, and (**D**) peak intensity of calcium signaling mediated by the interaction of integrin α_4_β_7_ with various concentrations of MAdCAM-1 under wall shear stress of 0.3 dyn/cm^2^. Data of each group are shown by boxplot with raw data point, where each horizontal line from top to bottom of a boxplot represents the maximum, upper quartile, median, lower quartile, and minimum for the group, respectively. At least 15 typical calcium signaling events of the firmly adhering cells should be collected among three repeat experiments for each group. Significant differences were shown by *p*-value, ns. for *p* > 0.05, **** for *p* < 0.0001.

Next, we evaluated the calcium response of RPMI 8226 cells that adhered to MAdCAM-1-coated substrates (2 and 20 μg/mL). The typical time course of calcium signaling showed that the concentration differences of MAdCAM-1 had a significant effect on the production of cellular calcium signaling (Figure 3A). As the density of immobilized MAdCAM-1 increased, the delay time for calcium signaling in RPMI 8226 cells decreased rapidly, indicating that enhanced interaction between integrin α_4_β_7_ and MAdCAM-1 hastened the cytosolic calcium release of RPMI 8226 cells (Figure 3B). Although no significant difference in peak time and peak intensity of calcium signaling was observed when the density of immobilized MAdCAM-1 increased, the peak time (Figure 3D) was shortened, and the peak intensity (Figure 3C) was increased to some extent. Taken together, firm arrest via the binding of integrin α_4_β_7_ and MAdCAM-1 was found to specifically trigger calcium signaling in RPMI 8226 cells, and the excitation rate of calcium signaling was dependent on the concentration of MAdCAM-1.

### 3.3. Shear Stresses Quickened and Enhanced Calcium Signaling of RPMI 8226 Cells

**Figure 4:**
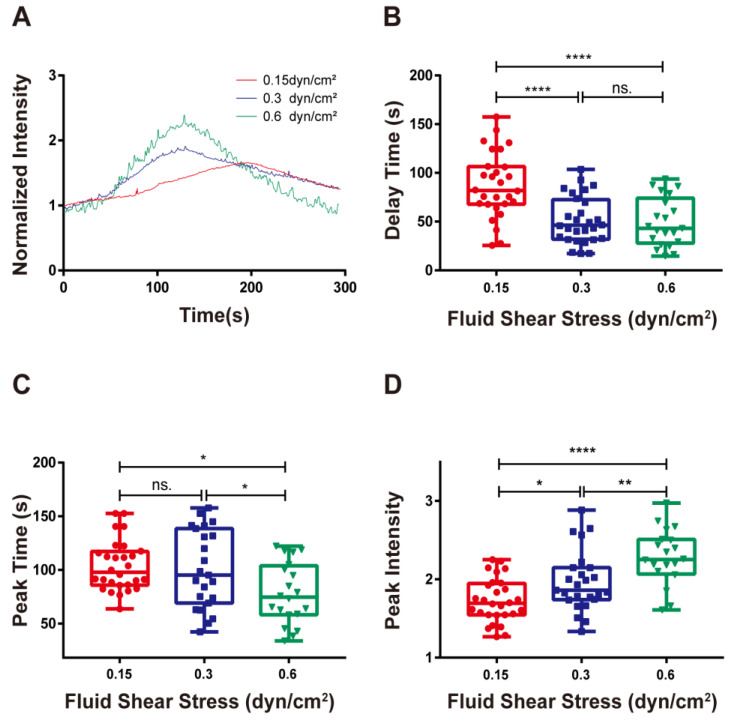
Force-regulated calcium signaling of firmly adhered RPMI 8226 cells. (**A**) Time course, (**B**) delay time, (**C**) peak time, and (**D**) peak intensity of calcium signaling for firmly adhered RPMI 8226 cells on the substrate coated with 10 μg/mL MAdCAM-1 under shear stresses of 0.15, 0.30, and 0.60 dyn/cm^2^. Data of each group were shown by boxplot with raw data point, where each horizontal line from top to bottom of a boxplot represents the maximum, upper quartile, median, lower quartile, and minimum for the group, respectively. At least 15 typical calcium signaling events of the firmly adhering cells should be collected among three repeat experiments for each group. Significant differences from the blank substrate group are shown by *p*-value, with ns. for *p* > 0.05, * for *p* < 0.05, ** for *p* < 0.01, and **** for *p* < 0.0001.

Previous studies have found that an external force is required for calcium signaling in neutrophil activation by integrin LFA-1. However, uncertainty remains regarding whether mechanical control can induce calcium signaling in lymphocytes by integrin α_4_β_7_ [16]. In this study, we evaluated the calcium response of RPMI 8226 cells that adhered to the 10 g/mL MAdCAM-1-coated bottoms under wall shear stresses of 0.15, 0.30, and 0.60 dyn/cm^2^. From the typical time courses of the calcium response, we found that shear stress enhanced the calcium bursting of RPMI 8226 induced by the binding of integrin α_4_β_7_ and MAdCAM-1 (Figure 4A). The delay in calcium signaling was reduced by increasing the wall shear stress (Figure 4B). Likewise, the peak time of calcium signaling decreased as the wall shear stress increased (Figure 4C). In contrast, the peak intensity, which was the highest amount of calcium signaling, increased with wall shear stress (Figure 4D). These results indicated that the calcium signaling of RPMI 8226 triggered by the interplay of integrin α_4_β_7_ and MAdCAM-1 was positively regulated by wall shear stress, in which the external force could accelerate the bursting speed of calcium signaling and enhance the strength of calcium signaling.

### 3.4. Integrin α_4_β_7_ Induced Calcium Influx Rather than Release into the Cytoplasm of RPMI 8226 Cells

**Figure 5:**
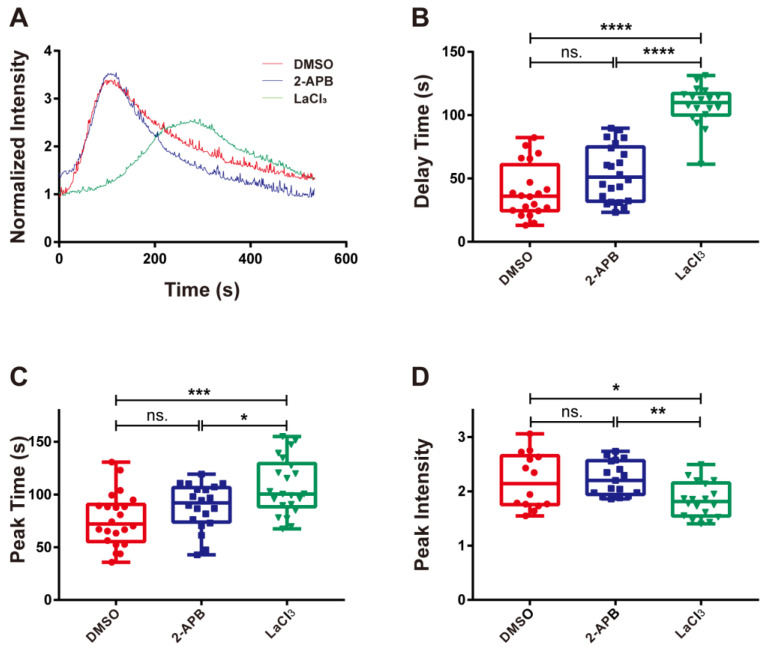
The effects of blocking the membrane calcium channel on calcium signaling. (**A**) Time course, (**B**) delay time, (**C**) peak time, and (**D**) peak intensity of integrin α4β7/MAdCAM-1 complex-induced calcium signaling of RPMI 8226 cells treated with the membrane calcium channel blocker LaCl_3_ and the IP3 inhibitor 2-APB or dimethyl sulfoxide (DMSO). The adhesion of RPMI 8226 cells was at a wall shear stress of 0.3 dyn/cm^2^. Data of each group were shown by boxplot with raw data point, wherein each horizontal line from top to bottom of a boxplot represents the maximum, upper quartile, median, lower quartile, and minimum for the group, respectively. At least 15 typical calcium signaling events of the firmly adhering cells should be collected among three repeat experiments for each group. The significant level of difference with the DMSO control group is shown by *p*-value, with ns. for *p* > 0.05, * for *p* < 0.05, ** for *p* < 0.01, *** for *p* < 0.001, and **** for *p* < 0.0001.

To determine whether calcium bursting was caused by cytosolic calcium release or extracellular calcium influx, 2-APB and LaCl_3_ were used to treat RPMI 8226 cells, respectively. Subsequently, the cells treated with different inhibitors were perfused into the flow chamber at a wall shear stress of 0.30 dyn/cm^2^. The cells were observed and recorded using fluorescence microscopy when they adhered to substrates coated with 10 μg/mL MAdCAM-1. The typical time courses showed that treatment with various inhibitors caused significant differences in the activation of calcium signaling (Figure 5A). Treatment with 2-APB had no significant influence on the delay time, peak time, or peak intensity, suggesting that calcium signaling in RPMI 8226 cells was unaffected by blocking the calcium channel on the endoplasmic reticulum with 2-APB (Figure 5B–D). However, treatment with LaCl_3_ significantly increased the delay and peak times and weakened the peak intensity (Figure 5B–D). In other words, calcium bursting in RPMI 8226 cells triggered by integrin α_4_β_7_ may be weakened and slowed down by inhibiting the calcium channel on the cellular membrane using LaCl_3_. These results indicate that calcium signaling induced by the interaction of integrin α_4_β_7_ and MAdCAM-1 mainly depends on the activation of calcium channels on the cellular membrane but does not require calcium release.

### 3.5. Kindlin-3 Was Involved in Calcium Signaling of RPMI 8226 Cells Triggered by the Integrin α_4_β_7_ in Flow

**Figure 6:**
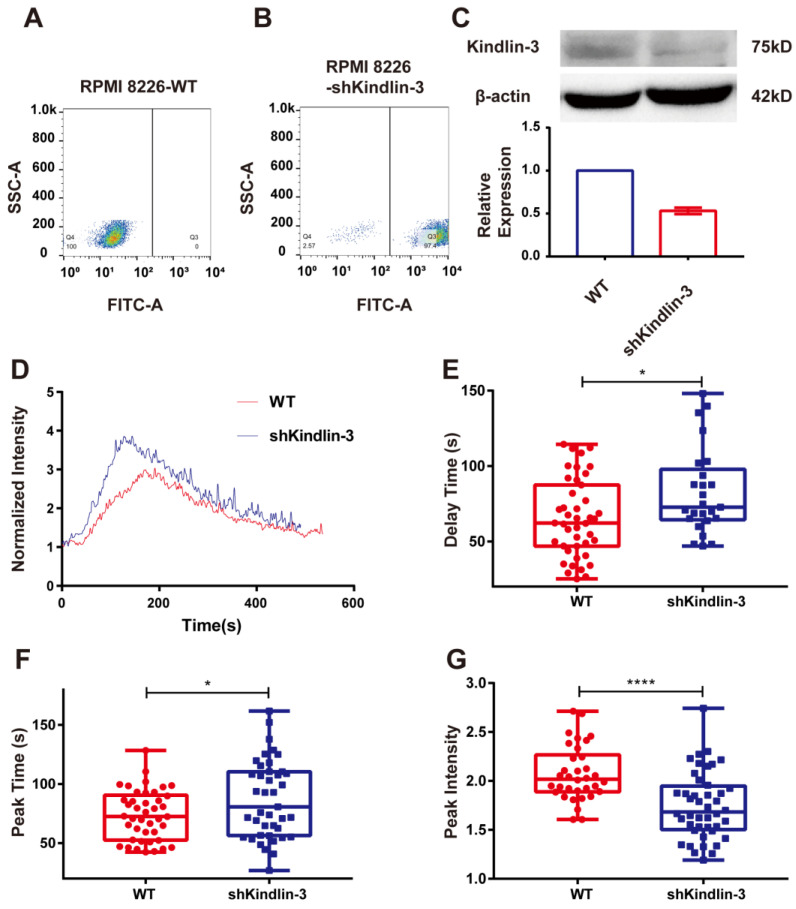
The effects of silencing Kindlin-3 on the calcium signaling of RPMI 8226 cells mediated by integrin α_4_β_7_/MAdCAM-1 complex in flow. (**A**,**B**) Positive rate of the RPMI 8226 cell line stably transfected by Kindlin-3 short hairpin RNA (shRNA) (RPMI 8226-shKindlin-3) compared to a non-transfected cell line (RPMI 8226-WT) identified by flow cytometry. (**C**) Western blotting verified the relative expression of total protein levels of Kindlin-3 between the RPMI 8226 cell line stably transfected by Kindlin-3 shRNA (shKindlin-3) and non-transfected one (WT). (**D**) Time course, (**E**) delay time, (**F**) peak time, and (**G**) peak intensity of integrin α_4_β_7_/MAdCAM-1 complex-induced calcium signaling of RPMI 8226 cells, with or without the silence of Kindlin-3, at a wall shear stress of 0.3 dyn/cm^2^. Data of each group were shown by boxplot with raw data points, where each horizontal line from top to bottom of a boxplot represents the maximum, upper quartile, median, lower quartile, and minimum for the group, respectively. At least 15 typical calcium signaling events of the firmly adhering cells should be collected among three repeat experiments for each group. Significant differences from the control group (non-treated cells) are shown by *p*-value, with * for *p* < 0.05 and **** for *p* < 0.0001.

Kindlin-3 is an important adaptor in integrin signal transduction. However, the mechanism by which Kindlin-3 assists integrin α_4_β_7_ to activate cytosolic calcium bursting remains unclear. To determine whether Kindlin-3 participates in the process of calcium signaling activated by integrin α_4_β_7_, we knocked down the expression of Kindlin-3 in RPMI 8226 and examined the calcium signaling induced by the interaction of integrin α_4_β_7_ and MAdCAM-1 under flow. The positive fraction of RPMI 8226 cells silenced by Kindlin-3 shRNA in the stably transfected cell lines was found to be >90%, as detected by flow cytometry after screening with puromycin (Figure 6A,B). At the same time, the immunoblotting of total lysates of RPMI 8226 cells with stable transfection of Kindlin-3 shRNA revealed a lower expression of Kindlin-3 compared with non-transfected RPMI 8226 cells (Figure 6C). The typical time course of Kindlin-3 knockdown cells showed a significant weakening of calcium signaling (Figure 6D). When compared to the control group, the knockdown of Kindlin-3 significantly lengthened the delay and peak times while weakening the peak intensity of calcium signaling in RPMI 8226 cells (Figure 6E–G), suggesting that the blockage of Kindlin-3 could disturb calcium bursting triggered by the interaction of integrin α_4_β_7_ and MAdCAM-1. Taken together, these results indicate that Kindlin-3 is involved in calcium signaling induced by the interaction between integrin α_4_β_7_ and MAdCAM-1.

## 4. Discussion

Integrin α_4_β_7_ facilitates the rolling, arrest, and subsequent activation of lymphocytes during the homing of circulating lymphocytes by interacting with MAdCAM-1 [3,4]. Previous studies have demonstrated that several integrins can induce calcium bursting in leukocytes [8]. For example, LFA-1 can activate calcium signaling after binding to ICAM-1 and activating the PLC-γ downstream signal pathway [24]. In addition, the interaction between integrin α_IIb_β_3_ and vWF can promote persistent calcium oscillations to maintain irreversible adhesion [25]. Likewise, calcium signaling in rat osteoclasts is activated by integrin α_v_β_3_ stimulated by its RGD (Arg-Gly-Asp) ligands, which are involved in osteoclast function [26]. However, the mechanism underlying the regulation of integrin-induced calcium signaling in lymphocytes remains unknown. In the present study, we evaluated the calcium bursting of lymphocytes mediated by integrin α_4_β_7_ binding with MAdCAM-1 under flow conditions. A parallel plate flow chamber, together with a fluorescent detection system, was used to perform the adhesion experiment. Furthermore, as a pattern cell of circulating lymphocytes, RPMI 8226 cells expressing integrin α_4_β_7_ were derived from a patient with plasmacytoma to simulate circulating lymphocytes functionally, which have been widely used in immunology research [20]. As such, our results using RPMI 8226 cells may reflect the physiological activity of human primary lymphocytes mediated by integrin α_4_β_7_ and MAdCAM-1. However, integrin α_4_β_7_ coupling with MAdCAM-1 to trigger the calcium response of RPMI 8226 cells in a process may be similar to calcium bursting triggered by P-selectin in neutrophils or HL-60 cells [11,12]. The fluorescence intensity of signaling activation has a typical plateau, a rising stage, and then a decreasing phase in firmly adhered cells. Nevertheless, there is a longer delay and peak time in integrin α_4_β_7_-mediated calcium signaling in RPMI 8226 cells than in the process of P-selectin. We speculated that the binding of integrin α_4_β_7_ to MAdCAM-1 triggered outside-in signaling and induced the global activation of integrin α_4_β_7_ in cells, triggering calcium signaling downstream [17,27]. This could be attributed to the longer delay time and peak time in calcium bursting of RPMI 8226 cells mediated by integrin α_4_β_7_.

The external force existing in the cellular microenvironment is crucial for the interaction of most biomacromolecules and cellular signal transduction [28]. Several mechanical techniques have been applied to investigate the effects of forces at the cellular and molecular levels, including parallel-plate flow chambers, atomic force microscopes, biomembrane force probes, and molecular dynamics simulations [29,30,31,32,33]. Using molecular dynamics simulations, the interaction of integrin α_v_β_3_ with its RGD ligands in a force-dependent process was demonstrated [33]. Similarly, the cleavage of vWF by ADAMTS-13 requires force-induced A2 unfolding during the process of hemostasis [34]. Previous studies have shown that HL-60 cells roll over E-selectin under the control of force-dependent bond dissociation. Therefore, the ideal shear threshold would ensure that the rolling is the most stable and regular [22]. These studies indicate that force is of great importance in the interaction of protein molecules. As for the mechanical regulation of cellular calcium signaling, the effect of external force on the calcium bursting of T cells is induced by the TCR-pMHC complex [14]. Similarly, Ca^2+^ entry through cation channels of megakaryocytic cells and human platelets is mediated by shear stress [35]. In addition, calcium signaling mediated by integrin LFA-1 binding to ICAM-1 is triggered and regulated by wall shear stress [15]. Importantly, Chateau et al. demonstrated that wall shear stress plays a key role in the cellular Ca^2+^-dependent rolling and Mg^2+^-dependent stable adhesion via integrin α_4_β_7_/MAdCAM-1 [36]. Recently, we found that shear stress firstly strengthened, then weakened, the tethering and rolling behaviors via integrin α_4_β_7_/MAdCAM-1 at a lower threshold of shear stress (also known as the “catch bond mechanism”). Based on these previous findings, herein, we further explored the effect of the external shear stress force on the integrin α_4_β_7_-mediated calcium response. Through quantitative measurements of peak time, delay time, and peak intensity, our results suggest that force may accelerate and intensify calcium signaling. This may be related to the catch bond mechanism, which regulates flow-enhanced roll and adhesion by reinforcing the engagement of integrin α_4_β_7_ with MAdCAM-1. This is similar to the activation of calcium signaling by the TCR/MHC interaction with force-governed affinity on T cells [14]. As a result, the integrin α_4_β_7_/MAdCAM-1 complex, as a force sensor, may trigger subsequent cytoplasmic signaling molecules and generate calcium signaling in RPMI 8226 cells.

Interestingly, we first demonstrated that calcium signaling mediated by integrin α_4_β_7_ was relevant to extracellular calcium influx but not intracellular calcium release. Thereafter, we observed that it was involved with Kindlin-3, the adaptor molecule binding directly to the cytoplasmic tail of the integrin β subunit. When lymphocytes are recruited to inflammatory regions, cytoplasmic Ca^2+^ acts as a signaling molecule to facilitate the transformation of the cell state from rolling to arrest to achieve functional activation [9]. During this process, the GPCR or P-selectin receptors of leukocytes first receive activated signals, and intracellular Ca^2+^ ions are released by activating the ER-related inositol 1,4,5-trisphosphate [10,12], known as “intracellular calcium release”. In addition, extracellular Ca^2+^ influx through the membrane calcium channel has been demonstrated to occur during leukocyte activation via the binding of integrin LFA-1 with ICAM-1 [15,16]. Similarly, we demonstrated that integrin α_4_β_7_-mediated calcium signaling is associated with extracellular Ca^2+^ influx, indicating that similar pathways are involved in the activation of calcium bursting through integrin α_4_β_7_. Additionally, Kindlin-3 in calcium signaling mediated by LFA-1 serves as a link in the formation of a focal adhesion complex combined with LFA-1 and receptor for activated C kinase 1 (RACK1), which is associated with the activation of ORAI-1, the main membrane calcium channel [16]. In addition to assisting the LFA-1-induced calcium signal, Kindlin-3 is required for the induction of the high-affinity conformation of LFA-1 through the engagement of PSGL-1 or CXCR2, which results in neutrophil arrest [37,38]. Compared with the assisting role of Kindlin-3 in LFA-1-mediated calcium signaling, Kindlin-3 may play a similar role in integrin α_4_β_7_-mediated calcium signaling. Unfortunately, Sun et al. suggest that Kindlin-3 dissociates from the tail of the β subunit in the CCL25-induction of the high affinity of integrin α_4_β_7_ induced by chemokine CCL25 [13]. These results indicate that Kindlin-3 may have different effects on the inside-out and outside-in pathways of integrin α_4_β_7_, similar to the negative role of Kindlin-3 in regulating the release of neutrophil extracellular traps [13,39].

**Figure 7:**
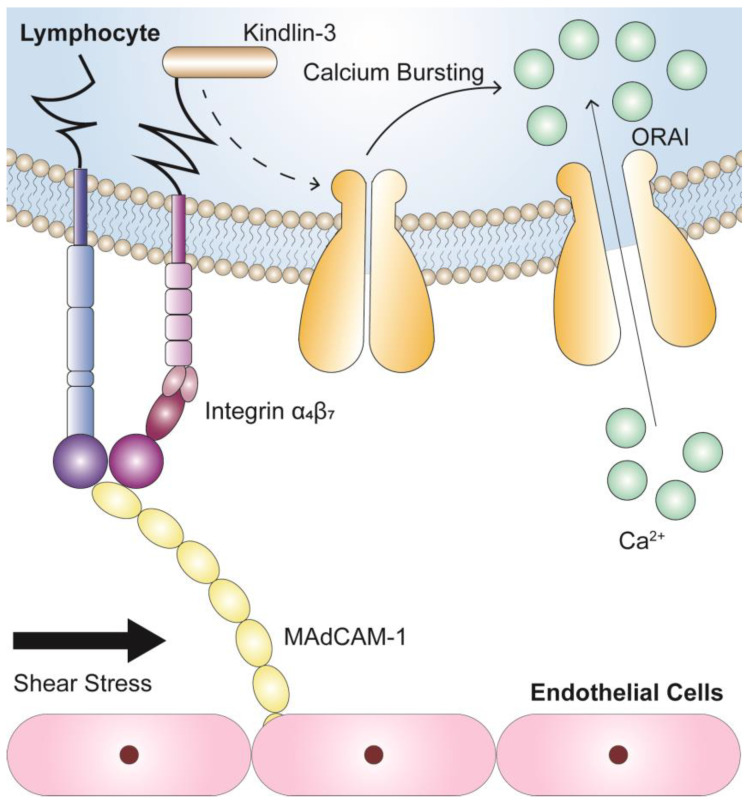
Potential mechanism of calcium signaling for circulating lymphocytes induced by the interaction between integrin α_4_β_7_ and MAdCAM-1. Integrin α_4_β_7_ engagement of MAdCAM-1 induces the force-dependent calcium signaling of firmly adhered RPMI 8226 cells in flow through a common pathway, in which Kindlin-3 is required, to subsequently activate calcium influx.

To summarize, we found that integrin α_4_β_7_ binding of MAdCAM-1 led to force-dependent calcium signaling in tightly adherent RPMI 8226 cells in flow via a pathway that required Kindlin-3 (Figure 7). These findings provide new insights into the mechano-chemical regulation mechanism for adhesion molecule-mediated intracellular signaling pathways, providing a basis for the development of novel concepts in risk assessment, clinical diagnosis, and the effectiveness of inflammatory disease and cancer treatment.

## Data Availability

Not applicable.

## Supplementary Materials

The following supporting information can be downloaded at: https://www.mdpi.com/article/10.3390/biom13040587/s1, Figure S1: Specific adhesion experiment; Figure S2: Adhesion probability of cells using different metal ion solutions; Figure S3: Adhesion frequency of integrin α_4_β_7_ specifically binding to MAdCAM-1 measured by AFM (atomic force microscope); Movie S1: Adhesion of RPMI 8226 cells to 20 μg/mL MAdCAM-1-coated substrate in flow chamber at a shear stress of 0.3 dyn/cm^2^; Movie S2: Calcium signaling of RPMI 8226 cells adhering to 20 μg/mL MAdCAM-1-coated substrate in flow chamber at a shear stress of 0.3 dyn/cm^2^.
