# Supplementary material for: Force-Regulated Calcium Signaling of Lymphoid Cell RPMI 8226 Mediated by Integrin α4β7/MAdCAM-1 in Flow
Source: Biomolecules. 2023 Mar 24;13(4):587. doi: 10.3390/biom13040587 (PMC10135767; doi:10.3390/biom13040587)
Supplement: Supplementary file 1 [file biomolecules-13-00587-s001.zip › biomolecules-2196690-supplementary.pdf]

Supplementary Files

Movie S1. Adhesion of RPMI 8226 cells to 20μg/mL MAdCAM-1-coated substrate in flow chamber at a shear stress of 0.3 dyn/cm^2.

Movie S2. Calcium signaling of RPMI 8226 cells adhering to 20μg/mL MAdCAM-1- coated substrate in flow chamber at a shear stress of 0.3 dyn/cm^2.

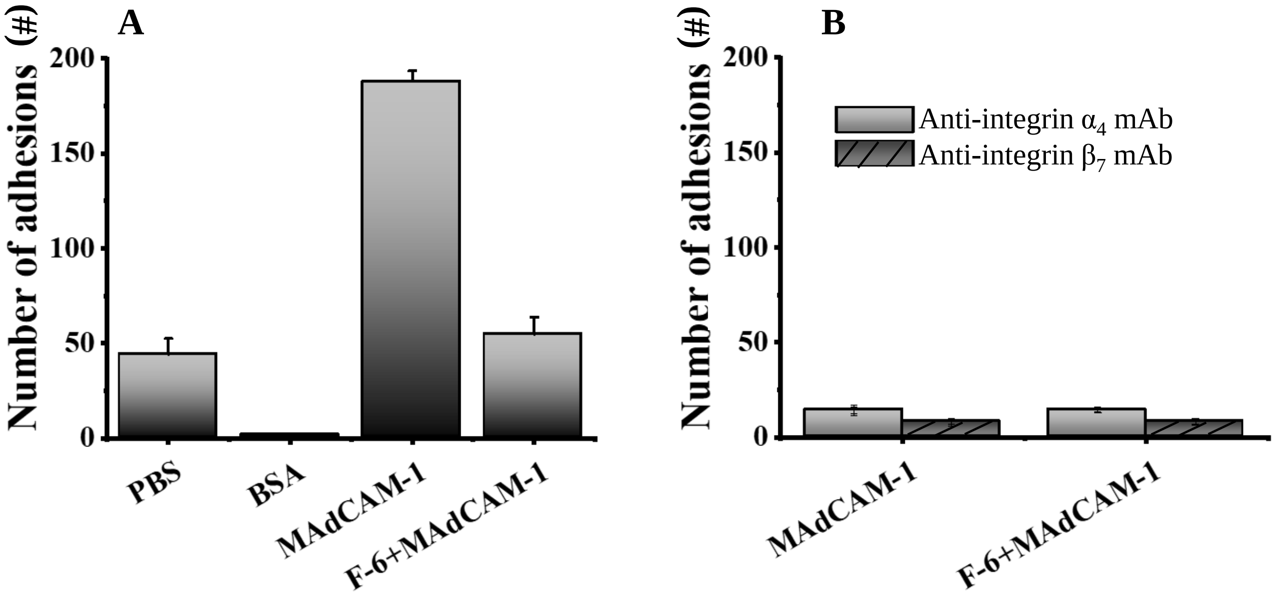

Figure S1. Specific adhesion experiment. (**A**) Adhesion blocking of MAdCAM-1. (**B**) Specific antibody blocking for integrin $\alpha_4\beta_7$. Adhesion number of integrin $\alpha_4\beta_7$-expressing RPMI 8226 cells (5 × 10^5/ml, suspended in HBSS with 2% BSA and 1 mM Ca^{2+}) adhered to MAdCAM-1 (2 μg/mL) coated substrates in a parallel-plate flow chamber at a particular shear stress (0.3 dyn/cm^2) for 1 min. For the negative control, 35-mm dishes were only coated with PBS or PBS with 2% BSA on the substrate of flow chamber, respectively. For the adhesion experiment, the dishes coated functional with MAdCAM-1 (2 μg/mL). For the blocking controls, the dishes were incubated with F-6 (anti-MAdCAM-1 blocking mAb), MAB1354 (anti-α_4 blocking mAb), and MAB4669 (anti-β_7 blocking mAb).

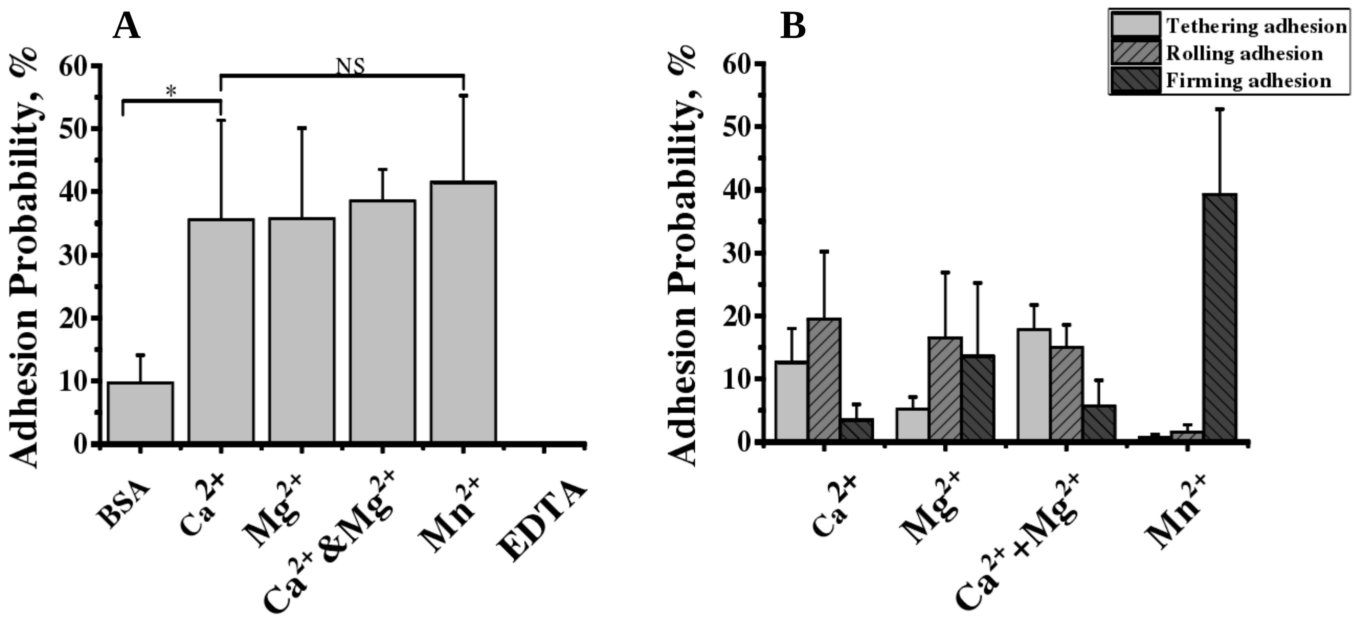

Figure S2. Adhesion probability of cells using different metal ion solutions. (**A**) Adhesion probability of cells. (**B**) Probability of different adhesion behaviors. The adhesion probability was calculated only for 1 min. Data represent the results of from three independent experiments.

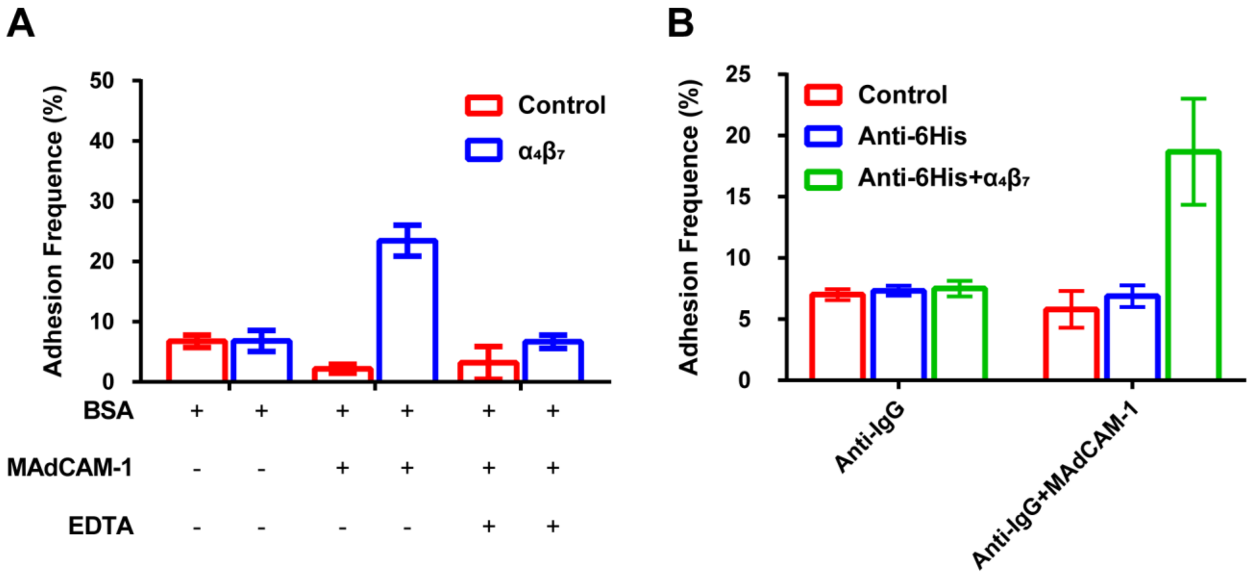

Figure S3. Adhesion frequency of integrin $\alpha_4\beta_7$ specifically binding to MAdCAM-1 measured by AFM (atomic force microscope). (**A**) Direct physical adsorption method. Adhesion frequency of integrin $\alpha_4\beta_7$ with the MAdCAM-1 coated with physical adsorption method. (**B**) Indirect antibody capture method. Adhesion frequency of integrin $\alpha_4\beta_7$ with MAdCAM-1 capturing with antibody. (Figure S3 in manuscript).
